# “As a psychiatry resident I am invited to explore my identity. But when I accept that invitation, I still encounter a wall.” A qualitative study on inclusion experienced by psychiatry residents with a migration background, sexual minority identity and/or working-class background

**DOI:** 10.1007/s10459-023-10236-9

**Published:** 2023-05-25

**Authors:** Donna Piëtra Muller, Petra Verdonk, Timotheüs Cornelis van de Grift, Mariken Beatrijs de Koning

**Affiliations:** 1 Arkin Mental Health Care, Amsterdam, the Netherlands; 2 Department of Ethics, Law & Humanities, Amsterdam UMC, location VUmc, APH Research Institute, Amsterdam, the Netherlands; 3 Zaans Medisch Centrum, Zaandam, the Netherlands; 4 Department of Psychiatry, Amsterdam University Medical Centre, location Academic Medical Centre, Amsterdam, The Netherlands

**Keywords:** Workplace inclusion, Psychiatry training, Sexual diversity, Migration background, Working Class, Assimilation

## Abstract

Diversity in terms of class, sexual identity and migration background among medical students in high income countries has increased greatly in recent decades. Some research into the experiences of these new groups of doctors has been performed. However, no previous research into the experiences of psychiatry residents specifically, is known. This qualitative study investigates how psychiatry residents, from these minoritized groups, experience their training regarding inclusion. Inclusion is defined as the degree to which one’s needs for connection and for being valued in one’s uniqueness, is satisfied. In-depth interviews with 16 psychiatry residents were conducted. These interviews were transcribed and coded using MaxQDA software. Initial themes that were constructed, were explored further in subsequent interviews and linked to literature. Finally, the developed themes were ordered in a model conceptualizing inclusion. Participants reported high belongingness within psychiatry training. Their experienced value in uniqueness, however, was generally quite low. Participants reported to experience little interest in and sensitivity for their perspectives and lived experiences from their co-workers. When faced with stigmatization and discrimination, participants reported lack of support from their colleagues. Assimilation was found to be the most frequently used coping strategy in dealing with diversity. Participants seemed to conform to the ‘neutral’ norm and experienced barriers in expressing themselves. Through this mechanism of assimilation, the added value that participants might bring with their unique knowledge and lived experiences was not used, both in patient care and in creating an inclusive climate within the organization. Moreover, assimilation is associated with psychological strain.

**Supplementary Information:**

The online version contains supplementary material available at 10.1007/s10459-023-10236-9.

## Introduction

### Diversity in undergraduate medical students’ demography and previous research

In recent decades diversity in migration background, and the openness about sexual orientation has increased in Dutch society (Kuyper et al., 2013; Leyerzapf, 2019). Simultaneously academic education has become more accessible to students from a working-class background (Thijssen et al., 2015). This has led to a growth in diversity among medical students and medical residents with regard to class, migration background, and sexual identities, although representation of people with a migration and/or working-class background remains low (Leyerzapf, 2019; Thijssen et al., 2015). Some research on the experiences of medical students from these minoritized groups has been conducted (Beagan, 2003, 2005; Leyerzapf, 2019) and studied experiences of working-class, LGBT students, and students from a migration background in Canada and found that these students find it hard to fit in. Leyerzapf (2019) found similar results when she interviewed medical students with a migration background in the Netherlands. She also found that doctors with a migration background are underrepresented in leadership positions and are less likely to become medical specialists. This causes underrepresentation of minoritized groups in key positions, where medical specialists from diverse backgrounds could function as role models and mentors.

Earlier studies in the last decades of the 20th century found a general tendency towards homogenization for all students during medical education (Haas & Shaffir, 1987; Shapiro, 1978). This tendency towards homogenization to a ‘neutral’ norm has also been found in studies that focus on students from these traditionally underrepresented groups (Beagan, 2000; Essed, 2005; Leyerzapf et al., 2018). This ‘neutral’ norm, amongst other things, consists of whiteness, heterosexuality and having an upper-class background (Essed, 2005; Razack, 2018).

### The unique position of psychiatry residents

Previous studies have mainly focused on experiences of students during undergraduate medical education. Less is known about the experiences of resident physicians in graduate medical education (Osseo-Asare et al., 2018). To our knowledge, no previous study in pursuit of mapping the experiences of psychiatry (or other) residents, from these minoritized groups, has been performed. We wonder if their experiences mirror the experiences described earlier. Several factors account for a unique position of psychiatry residents, and amount to the relevance of more knowledge on their experiences.

Following from their position, residents enjoy a higher status than interns, as they are further along their career path and have passed the selection for residency. Also, residents work in a specialization that appeals to them. Unlike interns, they have a work contract with a specific training institute and are part of the workforce. Residents occupy a unique position in having to balance learning and being supervised with the professional responsibility towards their patients (Sheehan et al., 2012). As future psychiatrists, psychiatry residents often operate in mental healthcare institutions in which hierarchical structures are less pronounced than in hospital settings. Furthermore, in the field of psychiatry, reflection on one´s position and how this affects one’s professional practice through learning therapy and intervision,^2^ is seen as essential and forms an integral part of the training program.

On the other hand, there is ample evidence that migration background, LGBT identity and low socioeconomical status are all associated with a higher incidence of several psychiatric diseases (Meyer, 2003; Muntaner et al., 2013). Minority stress (the cumulative effect of experiences with discrimination, expectations of rejection, internalized stigma and identity concealment) is argued to be a common underlying mechanism that makes individuals from these groups more at risk for mental illness (Meyer, 2003; Pascoe & Smart Richman, 2009). This, added to the fact that psychiatric diagnoses are highly stigmatized, might have an emotional impact on psychiatry residents from these groups, through identification with these patients.

### The concept of inclusion

Changing demographics and diversity policies in social institutions including higher education and health care increased diversity in the workforce at the end of the 20th century. Despite this increase in diversity, it turned out that minoritized individuals often did not feel at home in or able to contribute fully to their work organization. This led to the introduction of the concept of inclusion (Nivet, 2011; Shore et al., 2011). Where diversity describes numerical representation, inclusion describes how this diversity is dealt with.

In medical institutions, diversity and inclusion is deemed important. Employees who experience a high degree of inclusion show greater commitment to their organization, come to more creative solutions and remain associated with an organization for a longer period of time (Shore et al., 2011). A sense of inclusion invites multiple perspectives and experiences to be more openly shared and to inform thinking and practice. This consequently improves the quality of work (Conway-Hicks & de Groot, 2019; Shore et al., 2011).

Having a workforce consisting of people with a diverse background with regards to culture, gender and sexuality could aid in providing better care for patients from minorities, that are traditionally underserved (Beagan, 2000; Marrast et al., 2014), provided that these employees feel free to contribute to better understanding of patients from diverse backgrounds and to normalization of these identities (Nivet, 2011).

However, the process to reach inclusion does not come without difficulties: practices that were considered neutral and norms that provided guidance and identity are challenged, which can create discomfort and insecurity (Razack & Philibert, 2019). Many theories place the emphasis on institutional policies and practices (Zanoni & Janssens, 2007). Shore’s definition however centers the personal perspective and defines inclusion as the degree to which employees perceive that they are esteemed members of the work group through experiencing treatment that satisfies both their need for belongingness and value in uniqueness (Shore et al., 2011). Belongingness relates to a feeling of connection and acceptance which is also reflected in equal pay and professional opportunities. Uniqueness refers to individuals experiencing freedom to fully express themselves and to be valued for their unique perspectives. Shore’s definition therefore seems to fit best to this study, as it is based on the personal experiences of psychiatry residents as regards inclusion.

### Aim of the study

This qualitative study investigates how psychiatry residents from minoritized groups in terms of class, migration background and sexual orientation experience their training with regard to inclusion.

By combining the data from the interviews with the available literature, we aim to gain insight into the experienced inclusion and to identify mechanisms and factors that facilitate or obstruct inclusion.

We chose to study and combine the experiences of residents from these three groups, based on our focus on the mechanisms surrounding deviation from the norm. Focusing on a single identity could have provided an in-depth insight into the dynamics surrounding that particular identity but might have partly obscured underlying mechanisms. On top of this, by focusing on a specific category, mechanisms underlying in- and exclusion might be administered to the, supposed, characteristics of members of this group, obfuscating the universality of these mechanisms.

Our focus in this study is on the processes of inclusion and exclusion that cut across minoritized identities and therefore not primarily on intersectional analysis.

## Methods

### Research approach

**Fig. 1, (with permission):**
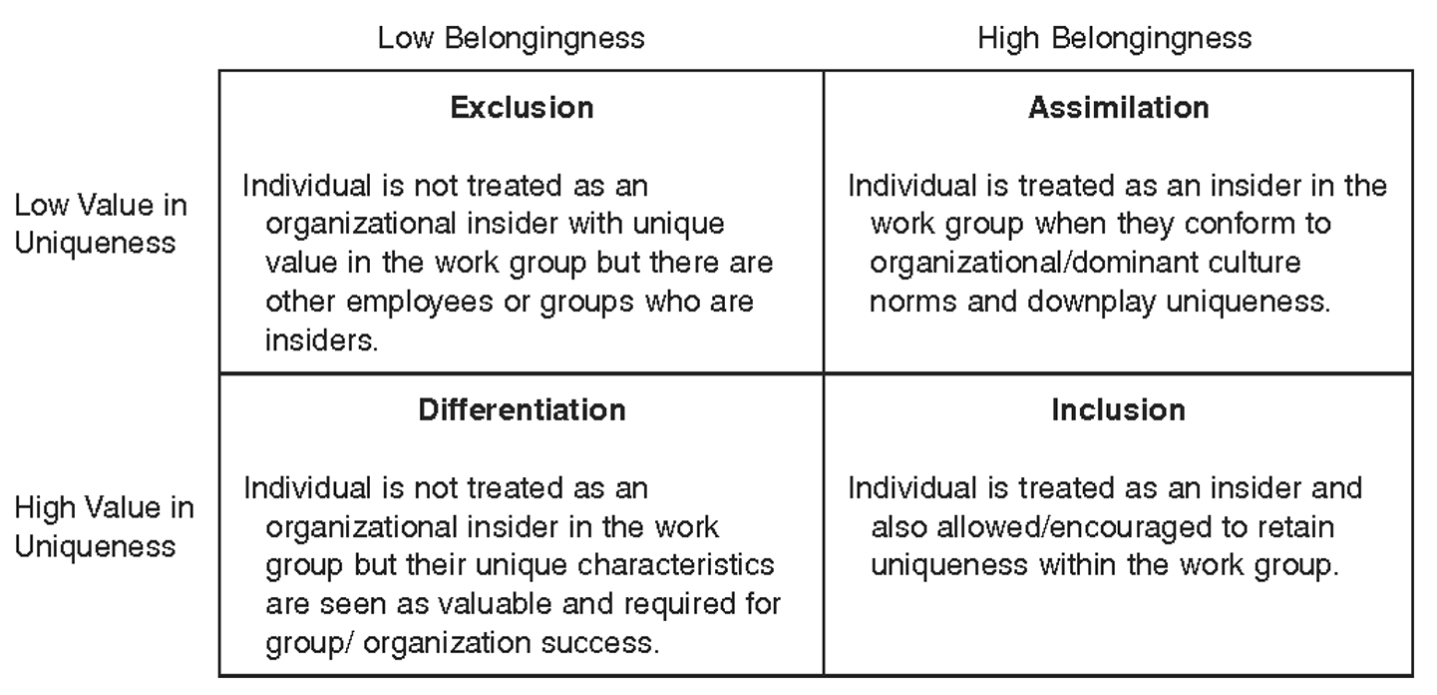
Inclusion framework (Shore et al., 2011)

This study, guided by a social constructivist approach, used qualitative, in-depth interviews with participants from several training institutes (Monrouxe & Rees, 2015). In this approach identities are not static but co-created through social interaction. Group composition, norms, and interactions influence how identities are constructed both internally and externally. In analyzing the data, we focused on interactions that made aspects of identity more or less salient and more or less comfortable. Since our study was based around the subjective experiences of the participants, we used reflexive thematic analysis to analyze the data (Braun & Clarke, 2022). The codes and themes that were developed in this analysis, were subsequently ordered in the model proposed by Shore (Shore et al., 2011) (see Fig. 1).

### Research team and reflexivity

The first author and interviewer, DM, is a female psychiatry resident of color. Both her parents were the first and only person in their families to have an academic education. She does not identify as heterosexual. From this position stems a sensitivity and alertness to the way in which people, with a migration background, a LGB identity and a working-class background are discussed and treated. A pitfall in coming from this position could be that experiences that resonated with the interviewer would be more salient to her than experiences that differed.

This study was closely supervised by MdK and PV. MdK is a female psychiatrist and researcher. She is white and identifies as heterosexual. Both her parents have a working-class background and were the only person in their families going to university. Although she has no working-class background herself, the background of her parents and their struggle made her sensitive to differences in socioeconomic status and their consequences. She has an interest in intersections with other underrepresented social categories. PV is a white female experienced researcher in the field of intersectionality in health and health care, with a background in psychology and gender studies in medicine. PV does not identify as heterosexual despite being in a long-term heterosexual relationship. Being highly educated and coming from a working-class background, she identifies with those who underwent the process of ‘socially upward mobility’. TvdG has contributed to the theoretical analyzes and writing. His position is that of a white cisgender homosexual researcher in the field of transgender care and a psychiatry resident.

### Participants and recruitment

Participants were recruited by email through the five training institutes in the greater Amsterdam area (the Netherlands). For reasons of purposeful sampling, an additional email was sent to the LGBT network of psychiatrists. Residents who were interested in participating were asked to send an e-mail to the interviewer (DM). Additional information on inclusion and exclusion criteria was collected through e-mail. Participants were provided with further information about the aim of the study, the privacy protocol, and gave their informed consent.

Inclusion criteria were that participants had to be in training for a year or longer (for reasons of work experience as residents) and to speak Dutch with (near) native fluency, since not speaking Dutch as a first language was considered to be a possible confounder. Participants had to self-identify with at least one of the following social identities: having a migration background, a LGBT identity and/or a working-class background. Labelling was based on self-identification, since the study focused on the experiences of the participants. Participants were interviewed about their understanding of these social categories as well as their identification with them. Coming from a working-class background could be defined in terms of parental education, income, or both. Considering migration background, no difference between heritage from a ‘Western’ or ‘non-Western’ country and between a first and second generation migration background was made in advance. For sexual orientation, self-identification was also used. Selection of study participants was done based on purposeful sampling.

No financial reward was given for participation. The Institutional Review Board of the AMC has declared that this study does not fall under the Dutch Law concerning medical research with humans, as the research question was not medical, and no intervention was applied (ID: W21_020 # 21.022).

### Data collection and analysis

Interviews started from a broad open question, asking about the experiences of the resident with regard to inclusion. Interviews were held with a flexible attitude towards the flow of the conversation, focusing on creating a safe environment. In this way the interviewer aimed to give optimal space for the experiences and emotions of the participants, in line with the reflexive thematic analytical approach (Braun & Clarke, 2022). A topic list (Appendix 1) was used to further explore residents’ experiences.

The duration of the interviews was 90 min on average (range 65–105 min). Due to covid restrictions interviews were partly conducted face-to-face and partly online. All interviews were conducted by DM, to enhance immersion and overview.

Interviews were audio recorded, ad-verbatim transcribed, and de-identified. The first three interviews were coded in parallel by a second coder, codes and initial themes were discussed, to optimize reflection. Next interviews were coded by DM, and themes were further developed. All authors met regularly to discuss coded data, to develop, review and refine themes, and to link themes to theory. The themes that were developed in this analysis, were subsequently ordered in the model as proposed by Shore (Shore et al., 2011). For coding and analyses MAXQDA software was used.

All participants have received the results of the study and were asked for feedback. There was no need for revision after feedback.

### Sample

Initially, no female resident with a non-heterosexual sexual orientation responded. Through the LGBT network of psychiatrists 3 additional female residents that identified on the spectrum between lesbian and bisexual, were recruited. No residents that identified as transgender reacted. Therefore, in the results section we will refer to the participants from the LGBT group as residents with a LGB identity. Twenty-nine residents in total responded to both calls. Selection of participants was done with the goal of having a diverse group of participants who could embody different perspectives in terms of gender, years of education, and (intersections of) studied minoritized identities. Even though the study aimed for a representative gender distribution, most participants identified as female, and none of the participants identified as nonbinary.

Table 1 shows an overview of the participants per subgroup or subgroups.

**Table 1:** Participants

Participant number	Gender	LGB	Migration background	Class difference
1	F	No	Yes	No
2	M	Yes	No	No
3	F	No	No	Yes
4	M	Yes	No	Yes
5	F	No	Yes	Yes
6	M	No	Yes	Yes
7	F	No	Yes	No
8	F	No	No	Yes
9	M	Yes	No	No
10	F	No	No	Yes
11	F	No	Yes	No
12	F	Yes	No	No
13	F	No	Yes	No
14	F	No	Yes	Yes
15	F	No	Yes	Yes
16	F	Yes	Yes	yes

Since the main researcher is a psychiatry resident, all 16 participants were close or distant colleagues. With three participants she had interacted in a more informal setting. Three other participants she had met during a shared course during the psychiatry training. With the other participants no relation prior to the study had been established.

## Results

In the interviews with participants from all three minoritized groups, the same overall themes surfaced. There were some subgroup differences in frequency of occurrence. These themes were analyzed through Shore’s model, which was found fitting for nearly all the main themes.

Most themes could be related to belongingness, value in uniqueness or assimilation, with assimilation being one of the four possible positions in the two-by-two matrix (Shore et al., 2011).

During the interviews, the theme ‘dynamics surrounding a patient with a shared minoritized background’ was developed. This theme does not fit in Shore’s model of inclusion, since there is a third party involved that breaks open the dynamic. This theme will therefore be discussed separately.

### Identification with minorized background

Some participants identified highly with their minoritized group membership. Others ‘felt identified’, they seemed to perceive this categorization more as something imposed on them. Most participants expressed changes in their awareness of and their identification with this part of their identity over time.

*My friends sometimes call me an Oreo. I am actually just Dutch. I am born here, and I think the Dutch culture is dominant in me. But I notice that as I’m getting older, I start seeing myself more in relation to my father. That my [ethnicity] side is getting a bit stronger in me. (No. 6; diff. in class/migration)*

*I am in a relationship with a woman. I think that makes me bisexual. I have had relationships with men in the past and I think I was in love with those men. But I must say that for me it is not really important how I label this aspect of myself. I think both [lesbian and bisexual] are fine. (No. 16; diff. in class/LGB/migration)*

*When you are from the working class, and you find yourself in a higher class… Well, it took me very long to discover, that I’m in a different class than where I belong. (NR 0.4; diff in class/LGB)*

### Belongingness

Participants from all three minoritized groups expressed feeling at home in psychiatry training and feeling to be part of the group. They often contrasted this with their feelings in general medical training. They often mentioned that psychiatry is a specialism where professionals are more used to and interested in people who are ‘different’.

*As a surgery intern, I felt very disadvantaged. I was not moldable into a typical female surgeon. I didn’t play hockey, so I couldn’t prove I’m a team player. I don’t play an instrument, which means I am manually disadvantaged. I was not a member in a fraternity, so what do I know about politics behind closed doors? I was too honest, too lesbian and too foreign. I always felt small. After that psychiatry felt like a warm bath.*

*(No. 16; diff. in class/LGB/migration)*

The choice of training institute also seemed to play a role. Participants with a migration background and participants from a working-class background mentioned that a sense of representation, and the feeling that an institute was not too elitist or academic, were reasons to apply to a general mental healthcare institution as opposed to an academic institute.

*With other institutions I had the feeling I had to be more academia minded […] and that I would have to join in with that white academic world. I deliberately chose not to do that.*

*(No. 5; diff. in class/migration)*

However, some instances were mentioned where the feeling of belonginess got jeopardized. This happened when the dominant culture did not match the values or customs that the participant grew up with. This could be related to differing in financial capital, cultural capital like knowing how to ski or sail, and understanding in-group humor and cultural norms. These differences could trigger feelings of insecurity and distance, as well as not knowing how to participate.

*There [country of ancestry] they easily find that you are quite present and here they say: “What do you think? Let your opinion be heard.” all the time. It seems like we should have an opinion about everything. Sometimes I just don’t have an opinion. But it is almost impossible not to have an opinion on something in the Netherlands. (No. 14; diff. in class/migration)*

*The sense of humor [in the resident group] is just a culture shock for me. I’m learning it. I can get it now. But in the core, it is not my humor, of course. It is totally different. So, I never know how to contribute to that kind of humor. (No. 5; diff. in class/migration)*

Most participants from a working-class background also reported feeling insecure with regards to the world of academic research and reported feeling ambiguous about the leading role in the hierarchy that they were expected to fulfil. Some participants with a working-class background mentioned that they felt insecure participating in discussions during education, because they felt less competent than their peers.

*I feel that I can’t join in with that level of ambition or entrepreneurship that is the norm in academia. Away from the framework, that I know from home, which is more left winged and safe and accustomed to working on a payroll. (No. 3; diff. in class)*

Participants with a working-class background seemed less conscious as regards their position than participants with a migration background or LGB identity. Rather, they expressed a sense of insecurity and distance towards academia and their co-workers, often without drawing a connection with their working-class background, and they tended to interpret the problems they experienced as ‘personal’. They often commented that reflecting on their class background as being member of a minoritized group helped them to better understand their experiences.

*I always fear to be found out, that I don’t belong. This feeling has only gotten worse as I advanced in my residency. I always feel that everyone is smarter than me. Everyone has more intelligent parents than me, everyone has had a more intelligent upbringing than me or something like that. So, everyone will do better and make smarter remarks than me. That is the feeling this training evokes in me. (No. 10; diff in class)*

Many participants reported a certain degree of representation to be crucial for a sense of belongingness. In environments with more representation they felt freer to express themselves, felt less watched and found opportunity to explore feelings that were related to their minoritized background. On top of this, role models from the same minoritized group could ignite a sense of pride and possibility for professional growth in participants. The degree of representation was generally considered low in absolute numbers by participants with a migration background and low in visibility by those with a LGB identity. Participants with a working-class background were less outspoken on the topic of representation.

*[Having a female psychiatrist with a Muslim background on a high position in the organization] That makes me feel that we are allowed to be here, that we are seen and valued. It also makes me suspicious: did she get this position because she wears a headscarf and is outspoken? But yes, it makes me proud. (No. 15; diff. in class/migration background)*

*Sometimes I worry if my patients will find me very gay and if that might influence the way they see me, what they share with me, or that they might even reject me. I would not know anyone with whom I could discuss this, who might have had a similar experience. (No. 9; diff. in LGB)*

In some instances, apparent representation could also jeopardize belongingness when participants felt classified to a group they did not feel connected to. The need to distinguish oneself from this group then arose.

*Amongst my study friends, there were also immigrants from lower social backgrounds. The so called “Tokkies’’ (Dutch slang for members of the ‘lower sociocultural’ class), who were bright enough to study. I didn’t feel a connection to them either. I am not like that. My parents are not from some small village in Morocco, they are from a Metropole, and I am born in a big city. (No. 13; diff. in migration background)*

#### Value in uniqueness

Positive value in uniqueness can be expressed through interest in personal experiences and knowledge and through sensitivity for one’s perspective as a member of a minoritized group. This value in uniqueness was generally perceived as low.

*I feel that the focus at the moment is very much that everyone is equal in the psychiatric training. I wonder if there is a lot of attention for the possible differences. (No. 2; diff. in LGB)*

In psychiatry training, personal perspectives and emotions are deemed important to discuss and explore. However, participants also stated that when expressing perspectives and emotions rooted in their minoritized social identities, they were regularly met with silence or unease.

*As a psychiatry resident I am invited to explore and develop my identity. But when I accept that invitation, I still encounter a wall. (No. 4; diff. in class/LGB/migration)*

Participants reported that little attention was given to differences, they were more often ignored or concealed. Participants reported being misidentified as belonging to the white, heterosexual upper-class norm. When colleagues knew that participants belonged to a minoritized group, they sometimes seemed to avoid this topic.

*At a certain moment my supervisor, who I thought knew me quite well, asked me, after a year of supervision: “do you have a girlfriend? “. I’m sure that I had mentioned my husband quite often. That hurt quite a bit. Because then there is very little attention for that deviant part of me. (No. 2; diff. in LGB)*

*The psychiatrist said to the patient “we might have a very different background than you.” This made me raise my eyebrows, but he didn’t see that. I thought “You should know what my background is. “(No. 5; diff. in class/migration))*

Several participants expressed that through their lived experiences they had developed a different perspective and sensitivity than usual. Topics that were personal to them were considered neutral by other colleagues. There seemed to be little awareness of this sensitivity and little room for their perspectives when topics related to diversity were discussed. This caused participants to feel unheard and alienated in these circumstances.

*They mentioned the quota of ´immigrant´ students and discussed whether all training programs had achieved the quota of 30%. I was shocked by that. I was the only person of color at the table, and I was shocked by the business-like way this was being discussed. That it apparently doesn’t mean a lot to other people… but to me it does. (No. 1; diff. in migration)*

In the curriculum, heterosexuality and whiteness also seemed to be the norm for patients while having an LGBT identity or a migration background seemed to be a red flag for psychopathology. All three male participants with a sexually diverse identity described the education courses within the training as heteronormative and often stigmatizing. This gave them a sense of ‘Otherness’ and made them reluctant to speak out. A few participants with a migration background also mentioned cases of stigmatization in the formal education course, but this seemed less salient.

*What I find difficult is that I know little about this theme in terms of actual psychiatric knowledge, but that I do have my personal experience. I think it would be easier if the education included learning about the construct of sexual orientation, gender identity, stigma and the psychiatric problems that are associated. That would make it easier for me to contribute, to be able to make the distinction between general knowledge and my personal experiences. (No. 2; diff. in LGB)*

*During this course I ask about the gender of the partner of a patient with sexual problems. The first reaction I get is: “Why do you ask that? It says that she is married and that she just gave birth a few months ago.” While one of my best [lesbian] friends had given birth to her daughter that week. That makes me angry. (No. 4; diff. in class/ LGB)*

#### Negative value in Uniqueness: stigmatization and discrimination

When uniqueness of participants was acknowledged, participants sometimes expressed that they felt to be reduced to this social identity in a way that tokenized or exoticized them. Several participants mentioned instances where they felt stigmatized or where clear discrimination was at play. This was most striking in interviews with participants with a Muslim background. In the theoretical framework proposed by Shore (2011), failure to recognize the worth of unique identities expressed through stigmatization (and ultimately discrimination), is seen as negative value in uniqueness.

*[Being asked by her supervisor if she had married through an arranged marriage:] That makes you feel, like you are a very small black immigrant. That people still think that this is very normal in my culture. It gives me the feeling that I must prove myself. But it has nothing to do with me. (No. 15; diff. in class/migration)*

*Sometimes people are curious and ask about all kinds of things about my homosexuality. There must be a balance between being interested in me as a person and some kind of voyeuristic curiosity. The fact that I am homosexual, doesn´t mean that I immediately just tell everything about that. (No. 2; diff. in LGB)*

More often participants mentioned witnessing stigmatization of patients with a similar minoritized background either directly or when talking about patients in team meetings. When this happened it often caused a sense of unsafety and a feeling of responsibility towards the patient. This was most apparent in the interviews with LGB participants, all of whom mentioned stigmatization of transgender patients. Several participants mentioned being sensitive to all forms of stigmatization of patients, because of their own experiences.

*The way patients from working-class neighborhoods are discussed feels devaluating. As if this is associated with all kinds of things like intelligence and emotional abilities and socio-economic status. For me that feels a bit like being put in a certain box. (No. 3; diff. in class)*

*I do think that colleagues have stigmatizing thoughts. “Here we have another dramatic borderline homosexual, with HIV, who is in crisis again and ‘suicidal’.” That about describes the stigma. I also find that stigmatizing. (No. 4; diff. in class/LGB)*

Several participants mentioned experiencing a lack of support from colleagues when faced with or addressing discrimination. Quite often the problem is downplayed or framed as a personal problem, even when the participant addresses stigmatization of a third person. There is no mention of supervisors or mentors taking action upon signals from participants. One participant expressed not knowing how to react when witnessing discrimination. Years later she still felt guilty for her lack of action. When participants— in exceptional cases—did get support, this created a sense of inclusion:

*A few times when I went into an isolation room for a consultation, the first thing that I heard was: “Dirty pervert, disgusting faggot.” Colleagues ignore that a bit. They disapprove of it. But there is little empathy; how was that for you, are you okay? […] I do miss that sometimes. (No. 4; diff. in class/LGB)*

*Sometimes a colleague makes a joke that is actually quite racist. When I react saying ‘that is actually not ok.’ people immediately say: ‘Don’t take it so personally. It is just a joke.’ But I think: “There is something in the core of this joke”. (No. 5; diff. in class/migration)*

*I found the fact that Muslim women were being referred to as ’women with headscarves,’ discriminating and haughty. When I discussed this with the team leader, he organized for me to talk to the person who made this remark. It wasn’t meant personally for me; I am not a Muslim. But anyhow. I had to talk to this lady. I didn’t feel supported at all […] After this experience I kind of closed myself off from these kinds of experiences. (No. 1; diff. in migration)*

*These days I get more support from people who have intervision with me. They have heard my previous frustration or someone else’s. This support is healing for me. (No. 4; diff. in class/LGB)*

### Assimilation

Many participants said that they adapted to fit the dominant norm. This could relate to choice of clothing, learning to ski or avoiding behavior that is seen as stereotypical for people from a certain ethnic group or with a certain sexual orientation. Sometimes participants saw these changes as enriching their identity, but more often participants expressed the feeling of becoming distanced from themselves.

*It [Homosexuality] is never explicitly a problem, but it is also never an asset, it is never a strength, that makes you say: tell me more about that. I notice that this makes me adjust to the norm. (No. 9; diff. in LGB)*

*That you are amongst people that are all dressed in suits, and you think, I am also all dressed up, but normally I just wear sneakers and jeans […|I can easily adapt and if I don’t pay attention, I drift away from myself; from who I am and what I want. (No. 3; diff. in class)*

Often participants from a working-class background experienced that what was needed to fit in as a psychiatry resident created distance towards their family and working-class background. They presented themselves differently in different contexts and sometimes felt they did not belong anywhere anymore.

*I have accepted that I will never belong anywhere for the full 100%. That I must be like a chameleon. The older I get, the harder I find it to be that chameleon. Because I must act differently amongst my fellow residents. I talk about different topics, do different things. Then I go back to my friends with a migration background that haven’t studied medicine. I talk differently with them, do other things with them. With my family again it is different. In the community where I come from it is different again. They are not highly educated. There, I must take care not to come across as too smart because than you are seen as arrogant. I keep having to adjust to the group of people I am interacting with at that moment. (No. 13; diff. in migration background)*

Participants also stated that they experienced different barriers in expressing themselves. Nearly all participants reported experiencing barriers to speak from their minoritized background. They feared being ignored, misunderstood or stigmatized or to create unease. Often participants described anxiety when they did speak out. This anxiety was less when in smaller groups, and in environments that were more diverse, which led to participants speaking out more in these settings.

*I recently told a few more colleagues that I grew up in a really poor family. That was very scary to do, but I am not ashamed of that anymore. There is no shame in that. I wanted to bring in my impoverished childhood, because I thought that if you can overcome your shame, there might be space for emancipation. (No. 4; diff. in class/LGB)*

### Dynamics surrounding patients with a shared minorized background

Nearly all participants stated that they felt they could add value in treatment of patients with similar backgrounds through their shared experiences and the trust these patients often emit towards them. Some participants stated that because of their personal experiences with ‘being (seen as) different,’ they could also empathize with patients from other minorized groups than their own.

*It helps when you recognize some of the pain of your patient, without having it consume you. I understand the feeling of displacement very well. The feeling of not having permission to be. I also understand that very well. Being expelled, not fitting in, not being allowed to be, because of characteristics you have not chosen for; I have experienced all of that. I think this makes me a better psychiatrist for these patients. (No. 16; diff. in class/LGB/migration)*

*My straight colleagues ask homosexual refugees questions that are a bit too blunt, like: when did you have your coming out? I prefer to ask: What did you tell your parents about why you are here? (No. 4; diff. in class/LGB)*

Some participants mentioned that, when treating a patient with a similar minoritized background, they became more aware of this part of their identity.

*With [colored] patients, I am more aware of the fact that I am also colored, because I hope that helps them to be a bit more open. So it is something which I… well, don’t mention obviously. But something that I am aware of and hope that it makes them more comfortable with me. So, that is interesting, actually, that I am aware of this [of my skin color] with patients, but not with my [white] supervisors, actually. (Nr 6; diff class/migration)*

Some participants also mentioned, when treating a patient with a shared minoritized background, they became more aware of differences in background and perspectives between them and the other residents and supervisors.

*I later noticed that people, who come from a family of doctors, who have more money, have another way of thinking. They can easily say that a patient can afford certain medication anyway. While I think: “No, some people really cannot afford that.” I also discuss these issues [financial problems] with my patients. Sometimes with colleagues, I notice that their background is different and that they talk very much from their own worldview. (Nr 12; diff. in class/LGB)*

The trust and recognition that the participants experienced from these patients could lead to the participants finding themselves in a bridging position between the patient on the one side, and the other practitioners on the other side. This position could create a sense of fulfilment but could also create tension.

*After completing an intake interview with a Moroccan patient, the attending psychiatrist, a white woman, was consulted. She attended to him in a way that made him feel uncomfortable and misunderstood. She had little time, and you could notice that she had preconceptions based on his background and asked questions from this perspective. When she left, the patient vented his dissatisfaction. He stated that he didn’t want to be attended to by this psychiatrist anymore. On the one hand, I could understand his feeling. On the other hand, it did not feel completely fair towards the psychiatrist, because she hadn’t had the chance to really work with him and show her competences. […] I felt quite overwhelmed to find myself in this situation. I was cautious in relating his feelings to the psychiatrist. I didn’t want to hurt her feelings, which made me search for words, but I did want to bring across his point of view clearly. (No. 5; diff. in class/migration)*

*It can also be a bit complicated, that you have to defend yourself or have to explain things. […] Whereas, with a supervisor that also has a different non-white ethnicity you do not have to explain these things, because he gets it, he has also experienced that and knows what it is about. (No. 1; diff. in migration)*

Participants quite often expressed unease in contributing their knowledge and experiences in team meetings. Several participants also mentioned a barrier to reflect with their supervisors on feelings provoked by their identification with patients with a shared minorized background and their understanding of their problems and emotions.

*I feel unease addressing those feelings [of recognition]. Even before I started my training, I found it very difficult to bring across the alikeness I felt with patients to my supervisors. (No. 5; diff. in class/migration)*

In some cases, being part of the same minoritized group as a patient could also be complicated because a special treatment was expected by the patient, boundaries were harder to maintain or expectations about sameness could not be met.

*A female patient [from same cultural group] in a manic state was admitted to the psychiatric ward where I worked. Her aunt came to visit, and we spoke about our background and how much authority your aunt has in our culture. The aunt then told me that she wanted to take the patient for a walk. The patient had not been granted permission yet to go outside. The aunt said: “It is going to be fine. I will bring her back. You know how it is, she respects me.” So, I granted her permission to go outside. Subsequently I was informed that the patient had ran away.*

*I realized that normally I would have discussed this with the psychiatrist, but that, because of our shared background, I thought that it would be ok. I now realize that the shared background also made it difficult for me to say: “No.”*

*I had to go back to the psychiatrist to explain that she had run away. But I did not explain what had happened. (Nr. 11; diff. in migration)*

*Sometimes I get the impression that they [patients with shared migration background] find it difficult that I am [from country of ancestry] as well, but that they still get the feeling I won’t understand them. Because I’m not that dark skinned and they think I’m half [country of ancestry], or because I am not from [suburb]. I feel that they can be disappointed, that you are not completely the same*. *(No. 1; diff. in migration)*

## Discussion

The study participants reported a strong sense of belongingness within the psychiatry training. They however experienced little interest, appreciation, and protection for their uniqueness. According to Shore, the found combination of relatively high belongingness and low value in uniqueness points to assimilation as the most common coping strategy. Assimilation is defined here as a position where individuals are treated as insiders in the work group when they conform to the dominant norms within the organization and downplay their uniqueness (Shore et al., 2011). A tendency towards assimilation was also found in this study. Participants seemed to adjust to the norm and to experience barriers in expressing themselves, when they wanted to voice different perspectives.

### Assimilation in medicine and psychiatry

The tendency toward assimilation is well-documented in undergraduate medical training. It is argued that the ideal of the ‘neutral and scientifically objective doctor’, who does not have any (deviant) social characteristics, plays a role. These characteristics are considered something private, that should not play a role in one’s profession. Through this paradigm of neutrality, the influence of individual characteristics is denied. This enforces the white, heterosexual, and upper-class norm, marking other perspectives as less neutral and objective and therefore less valid (Beagan, 2000; Essed, 2005; Haas & Shaffir, 1987; Leyerzapf et al., 2018; Shapiro, 1978).

Psychiatry is gradually shifting away from the (impossible) ideal of the neutral psychiatrist to awareness of and reflection on the role of the personal perspective of the psychiatrist in one’s practice. This reflection is mainly apparent in the training all psychiatry residents receive in psychotherapy practice and in building and maintaining a therapeutic relationship (Psychopathology Committee of the Group for the Advancement of Psychiatry, 2001; Summers, 2018). The study participants also expressed feeling more welcomed and accepted in psychiatry. They expressed experiencing less discrimination and exclusion than before, during their internships. For some participants, the perceived openness in psychiatry had played a role in their specialty choice. In an online survey on specialty choice among sexual and gender minorities in the United States, with 358 respondents -residents and practitioners- from these minorities, perceived inclusivity seemed to play an important role in specialty choice as well. Psychiatry was rated as the specialty with the highest perceived inclusion (Sitkin & Pachankis, 2016).

Still participants faced a lack of interest in and sensitivity for their unique perspectives. The fact that all psychiatry residents and their mentors went through the same homogenizing process of undergraduate medical training might play a role. In addition, participants mentioned pathologizing of their minoritized backgrounds in formal education and stigmatization of patients with similar backgrounds. These experiences made participants reluctant to draw attention to this part of their identity.

### Assimilation accounts for invisibility and isolation

The strategy of assimilation makes the presence of residents from minoritized groups in the profession less visible. As a consequence, the workforce seems more homogeneous than it in fact is. This dynamic is also found in Beagan’s (2005) research among medical students in Canada. Despite the fact that almost all interviewees in her study insisted that there were no working-class students in the school, about 15% of students identified as coming from a working-class or impoverished family background, while an additional quarter to a third came from lower to middle-class backgrounds (Beagan, 2005). This mechanism of invisibility may cause residents from with a minoritized background to feel more isolated than necessary and to experience a greater lack of role models than actually necessary. Potential role models may also feel barriers to express parts of their identity. Through this mechanism, alliances are not easily made, and power dynamics are obfuscated. This causes the ‘diversity debate’ within the organization to stagnate and causes discomfort in dealing with diversity. The norms, professional self-image, and shared knowledge within the group all become more immutable than the actual group composition justifies. Moreover, assimilation is associated with negative self-directed emotions, feelings of social exclusion and emotional exhaustion (Shore et al., 2011). This tendency towards assimilation was found in nearly all participants. The mechanism of conforming to the neutral norm is also found in several other studies on diversity and inclusion in medical education (Beagan, 2000; Essed, 2005; Leyerzapf et al., 2018).

### Assimilation in relation to patient care

Assimilation as a coping strategy seems less effective when treating patients from the same minoritized group. Participants often felt that they could be of added value in these treatments, a result that is also found in two fairly recent studies on experiences of health professionals from lower class origin (Beagan et al., 2022; Conway-Hicks & de Groot, 2019). Participants however mentioned a barrier to share their knowledge, experiences and emotions that stemmed from belonging to a minoritized group in team meetings, intervision and supervision settings. This hesitation is also described in research about interethnic supervision dyads (White-Davis et al., 2016). On a personal level, this gives participants little opportunity to explore and develop this part of their professional identity and to improve the way they use their identity as a tool in contact with their patients. Related to the treatment team and the profession as a whole, through this barrier, an increase in diversity in psychiatry residents does not directly lead to a deeper understanding of the possible processes surrounding the psychopathology of these patients. Finally the professional ideal of the objective and neutral doctor is not challenged, when participants do not show their minoritized identities and the added value these identities have through their unique knowledge and lived experiences. Hereby, they do not add diversity to the idea of ‘the doctor’ as a person who might have minoritized identities, which leaves these identities as categories only applicable to patients. As a consequence, the potential that these residents have in challenging the binary between the ‘normal doctor’ who has ‘no or only privileged social identities’ and the ‘pathological patient’ who does have minoritized identities is not used, and these identities remain pathologized.

### Applicability of Shore’s framework for inclusion

We applied the model of Shore to the themes that we identified from the interviews. Shore’s model was useful for analyzing the relationship between participants and coworkers in various contexts of the psychiatry training. However, it appeared to be less applicable to analyzing the dynamics that involved a patient with a shared minorized background.

Shores model describes bilateral relationships between a dominant group and individuals who might or might not feel included. In the dynamics involving a patient from the same minoritized group, a triangular relationship is at play in which participants must balance their need for inclusion in the medical team with their need for inclusion in the social group that is shared with the patient. As the residents are positioned in the middle of the doctor-patient binary, assimilation to one side may create distance towards the other side. To understand the position and dilemmas faced by the participants, both the relation with the professional group and with the patient should be included in the model. By using a model that includes both relations, the socially constructed distance between the identity as psychiatry resident and the minoritized identity shared with the patient is also exposed.

### Contextualization of class

All participants described a process during their training of how to deal with their identity in their professional practice. Many participants with a migration background and/or an LBG identity seemed to be engaged in a conscious process of reflection on their identity and how this influenced their role as a professional. Emancipatory movements surrounding these identities may stimulate and facilitate this reflection. In Dutch society, class inequalities are generally ignored and often denied (Thijssen et al., 2015; van Eijk, 2013). This may explain the found difficulties participants from a working-class background have in contextualizing themselves and their experiences, choosing instead to label these experiences as ‘personal’. Participants from a working-class background however, often described the same experiences and emotions: many stated to experience a sense of insecurity and distance towards academia and their coworkers. They also described a sense of displacement both in academia and their system of origin. These themes are also described in the literature on students from a working-class background (Beagan, 2005; Thijssen et al., 2015).

### Moving from assimilation to inclusion

To transition from assimilation to inclusion, an increase of the value given to minoritized identities is necessary. It asks for interest in and protection of these identities by mentors, supervisors and other co-workers, which in turn leads to greater openness about and better use and development of these identities.

In our study participants also mentioned instances where they did experience interest in and protection of their minoritized identities and felt free to express emotions and perspectives stemming from these identities.

Being supervised or mentored by a role model with a similar background was often mentioned as a positive experience, where reflection on this part of their identity happened automatically and participants felt supported. The importance of visible diversity in mentorship and role models is also mentioned in several studies on educational experiences of health professionals from minoritized groups (Claridge et al., 2018; Leyerzapf et al., 2018; Marrast et al., 2014; White-Davis et al., 2016; Wyatt et al., 2022). A study on a LGBTQ + paired mentorship program in Canada showed that role-modeling by LGBTQ + physicians provided reassurance to trainees about the capacity to achieve personal success, and promoted a sense of belonging amongst peers and faculty (Beanlands et al., 2020).

In our study, some participants felt that an open attitude from a supervisor, regardless of shared identity, was sufficient for them to feel free to share emotions and experiences stemming from their minoritized identities. Literature on interethnic supervision dyads suggest that proactivity in addressing diversity by supervisors and mentors and an attitude of cultural humility can help to facilitate sharing perspectives and emotions that stem from belonging to a minoritized group (Berg & Wright-Buckley, 1988; Burkard et al., 2006; Constantine & Kwan, 2003).

Participants mentioned to find it easier to express themselves and find allies in smaller groups and in groups where they experienced more representation. Several studies on inclusion in medical education have shown that creating bonds with like-minded people, both with and without shared minoritized backgrounds, helped to create a sense of community and to gain peer support (Conway-Hicks & de Groot, 2019; Pride et al., 2022; Steele, 1997; Verdonk et al., 2021).

Participants often expressed reluctance to report stigmatization and discrimination. In the few instances where they did speak up at work, participants mentioned that they hardly found support. This was also found in previous research on experiences of minority residents (Osseo-Asare et al., 2018). Studies suggest that bystanders such as co-workers, mentors and supervisors are often uncertain of the correct response when confronted with acts of stigmatization or discrimination. Anticipatory discussions to think through and discuss possible reactions are advised. This could take place during supervision and team intervision or in meetings specifically set up for this purpose. Thinking through and discussing possible reactions can reduce the feeling of being overwhelmed in the moment. Effective bystander responses should acknowledge student preferences, and, when a patient is involved, incorporate patient context and the interpersonal dynamics in the room (Bullock et al., 2021; Verdonk et al., 2021).

### Strengths and Limitations

#### Strengths

To our knowledge, this is the first study which examines inclusion in psychiatry residents and aims to contribute to a better understanding of the mechanisms at play. By focusing on three different minoritized groups and analyzing them together, common mechanisms as well us unique aspects could be identified that could be considered to be related to belonging to a minoritized group. The main researcher comes from a similar position as the participants, being a psychiatry resident and belonging to an intersection of multiple categories of difference. This facilitated access to and trust from the study population. This has contributed to the gathering of rich data describing complex, potentially shame-laden interactions this study is based on.

#### Limitations

The participants in this study were recruited based on their reaction to a call for participants. It is possible that those who were more than average interested in diversity and inclusion responded. All participants are psychiatry residents, which might be of influence in their positive evaluation of psychiatry. Whether their experiences mimic or are contrasted by experiences from residents from other specializations is unknown, since to our knowledge no other study of experienced inclusion in residents has been conducted. Furthermore, considering the limited amount of 16 participants in this study, findings have to be taken with caution. Although all participants describe similar experiences with regards to the mechanisms of inclusion and assimilation, further research is required to explore the differences between the different groups and intersections of these groups. All participants are from different training institutes in the greater Amsterdam area. Whether participants in less urbanized areas in the Netherlands have similar experiences cannot be concluded from this study. Because of this, assumptions and generalizations based on the current study should be made cautiously.

### Recommendations

The themes identified in this study are largely similar across the three minoritized groups. Focusing on the needs of individual students from specific minority groups is therefore ineffective. Rather than pushing residents from specific minoritized groups to assimilate, efforts should be aimed at valuing and providing opportunities to share and reflect on their unique perspectives and experiences. Interventions should focus on:

- Increasing diversity and visibility of potential role-models. Creating a safe space to explore and develop one’s professional identity, through supervision, intervision and peer support networks for residents from with minoritized backgrounds;
- Increasing sensitivity for and interest in the perspective of colleagues with minoritized backgrounds, when discussing patients or other issues related to diversity. Teaching about diversity and the psychological effects of belonging to a minority in the formal curriculum, while paying attention to not making diversity a cue for (psycho)pathology;
- Taking adequate action upon stigmatization and discrimination by all members of the workforce at every level of the organization. Lowering barriers for reporting stigmatization and discrimination;
- Improving contextualization and visibility for residents from the working-class especially, through peer support groups and/ or mentorship programs, given the lack of awareness of the dynamics surrounding this category;
- Stimulating co-workers from both minoritized and non-minoritized groups to reflect on their social positioning and lived experiences and how these influence their professional practice.
- Since this study is the first to map the experiences of psychiatry residents from the three mentioned categories of difference, further study into these mechanisms and ways of altering them is needed. Mapping the experiences and perspectives of non-minoritized co-workers with regard to inclusion could be a next step in understanding the dynamics of the interactions.

## Conclusion

Participants reported a strong belongingness within psychiatry training. This contrasted strongly with their experiences in general medical training. The experienced value in uniqueness, however, was generally quite low. Participants reported that they generally experienced little interest in and sensitivity for their perspectives and lived experiences as minoritized group members. When faced with stigmatization and discrimination, participants often reported lack of support from their colleagues, which could jeopardize their sense of inclusion within the organization. Assimilation was found to be the most frequently used coping strategy in dealing with diversity. Participants seemed to conform to the ‘neutral’ norm and to experience a barrier in expressing themselves. Through this mechanism, the added value that participants might bring with their unique knowledge and lived experiences is not used in patient care, in challenging the ‘neutral’ identity of the doctor and in creating an inclusive climate within the organization. Moreover, assimilation is associated with psychological strain. Increased inclusion could also help in creating a space conductive to exploring and developing one’s identity as a psychiatrist. Psychiatry is a profession wherein the subjective emotions that are triggered by the patient play an eminent role in understanding, diagnosing, and treating the patient. Psychiatry also deals with patients that struggle from the consequences of belonging to a minoritized group. For an adequate treatment of these patients, an open dialogue about the identity of psychiatry residents is needed.

### Electronic supplementary material

Below is the link to the electronic supplementary material.

Supplementary Material 1

## Footnotes

2 Intervision refers to a peer led, non-hierarchical reflection method, where complex cases are brought in, accompanied with the request for reflection from the Intervision group. In these meetings colleagues can reflect on their interaction with the patient, find emotional support and share good practices. These meetings are part of the professional practice of psychiatrists and other mental healthcare professionals.
