# Supplementary material for: “As a psychiatry resident I am invited to explore my identity. But when I accept that invitation, I still encounter a wall.” A qualitative study on inclusion experienced by psychiatry residents with a migration background, sexual minority identity and/or working-class background
Source: Adv Health Sci Educ Theory Pract. 2023 May 25;28(5):1633–56. doi: 10.1007/s10459-023-10236-9 (PMC10700411; doi:10.1007/s10459-023-10236-9)
Supplement: Supplementary file 1 — Supplementary Material 1 [file 10459_2023_10236_MOESM1_ESM.docx]

**Topic list**

Age:

Gender:

Level of education of parents:

Migration background:

Sexual orientation:

Training institute:

Year of training:

I would like to learn more about your experiences with regard to inclusion or a lack thereof in the psychiatry training. With psychiatry training we mean everything you encounter as a psychiatry resident, during formal training, on the work floor, during congresses, socializing with other trainees etc. Could you tell me about these experiences and describe them as concrete as possible?

Topics:

- Belonging
  - Feeling part of the group of residents, the workforce etc.
  - Feeling different from colleagues
  - Being perceived as/ made into the ‘Other’
- Fitting in
  - Fitting in the norm
  - Having cultural capital that is the same/different
  - Having social capital that is the same/different
- Being heard
  - Being heard
  - Feeling understood
  - Speaking out
  - Reactions on speaking out
  - Feeling secure/insecure
- Colleagues
  - Effect of representation
  - Identification/role models
  - Being a role model
- Patients
  - Patients with whom you can identify
  - Reflection on identification
  - Dynamics in the team/ interaction with supervisor when treating a patient with whom you can identify.
  - Stigmatization of patients
- Formal education/case studies
  - Cases with patients from the same category of difference
  - Knowledge offered
- Moments of inclusion
  - Sense of being seen with your individual qualities
  - Sense of being part of the group
- System of origin
  - Match between norms and culture of system of origin and psychiatry training
  - Changing identity during psychiatry training
  - Changing relation to system of origin
